# Redefining Access to the Mesiotemporal Lobe: The Transplanum Polare Approach with Cadaveric and Operative Video Demonstration

**DOI:** 10.3390/brainsci16040351

**Published:** 2026-03-25

**Authors:** Jesse Shamsul, Alessandro Pesaresi, Daniele Starnoni, Samia Messaoudi, Lorenzo Dolci, Hugues Cadas, Sami Schranz, Sara Sabatasso, Vincent Dunet, Roy T. Daniel, Pablo González-López, Lorenzo Giammattei

**Affiliations:** 1 Department of Neurosurgery, Lausanne University Hospital, CH-1011 Lausanne, Switzerlandalessandro.pesaresi@unito.it (A.P.); daniele.starnoni@chuv.ch (D.S.);; 2 Neurosurgery Unit, Department of Neuroscience “Rita Levi Montalcini”, AOU Città della Salute e della Scienza di Torino University Hospital, University of Turin, 10126 Turin, Italy; 3 Faculty of Biology and Medicine, University of Lausanne, CH-1005 Lausanne, Switzerland; 4 Unité Facultaire d’Anatomie et de Morphologie (UFAM), University Center of Legal Medicine Lausanne-Geneva (CURML), Lausanne University Hospital, CH-1011 Lausanne, Switzerland; 5 Neuroradiology Unit, Service of Diagnostic and Interventional Radiology, Department of Medical Radiology, Lausanne University Hospital, CH-1011 Lausanne, Switzerland; vincent.dunet@chuv.ch; 6 Department of Neurosurgery, Hospital General Universitario Alicante, 03010 Alicante, Spain; 7 Department of Optics, Pharmacology and Anatomy, University of Alicante, 03690 Alicante, Spain

**Keywords:** temporal lobe, planum polare, amygdala, hyppocampus, Sylvian fissure, transtemporal approach

## Abstract

**Objectives:** This study aims to define the surgical anatomy, technical feasibility, advantages, and limitations of the TPPA through detailed cadaveric dissection and a representative clinical case, evaluating its potential as a safe and effective alternative to traditional approaches to the mesiotemporal lobe. **Methods:** A cadaveric dissection was performed on one adult head injected with colored latex, using standard microsurgical instruments and high-definition video documentation. Each procedural step was recorded and illustrated with cadaveric photographs. Additionally, a clinical case of mesiotemporal cavernous hemangioma resected via TPPA is presented, including an operative video. **Results:** The dissection demonstrated a direct and safe trajectory to the amygdala and hippocampal head, with clear identification of key vascular and white matter landmarks. In the clinical case, the lesion was completely resected with no postoperative neurological deficits. **Conclusions:** The TPPA represents a novel microsurgical corridor to the mesiotemporal region, minimizing cortical disruption, Sylvian fissure dissection, and manipulation of middle cerebral artery branches. Although its exposure is limited posteriorly, the TPPA could offer an optimal balance between functional preservation and surgical accessibility, constituting a valuable addition to the modern microsurgical armamentarium.

## 1. Introduction

The mesiotemporal region is one of the most challenging areas to access due to its proximity to critical neurovascular structures [1,2,3]. Several approaches have been described, including transcorticall [4,5], subtemporal [6,7], transsylvian [8], supracerebellar transtentorial [9], endoscopic endonasal, and transorbital corridors [10,11,12], each with specific advantages but also notable limitations. The transplanum polare approach (TPPA) has recently emerged as a promising alternative, offering a direct anterobasal route to the amygdala and hippocampal head while minimizing Sylvian fissure dissection and avoiding cortical transgression [13]. Nevertheless, its surgical anatomy and technical nuances remain incompletely characterized. The aim of this study is to present a stepwise cadaveric dissection and an illustrative clinical case to clarify the anatomical rationale, technical feasibility, potential advantages and limitations of the TPPA.

## 2. Materials and Methods

The anatomical dissection was performed at the Neurosurgical Education and Training Laboratory (NET Lab) of the Lausanne University Hospital, Switzerland. The surgical anatomy of the transplanum polare approach was described using 1 adult cadaveric head (male; aged 79 years) injected with colored latex, non-formalin-fixed. The procedures were performed with standard microsurgical instruments including a high-speed drill (Midas Rex; Medtronic, Minneapolis, MN, USA), surgical microscope (Leica Microsystem, Wetzlar, Germany) and a high-definition camera (Karl Storz GmbH, KG, Tuttlingen, Germany). The dissection video was recorded using a 2D/4 K camera (Karl Storz GmbH, KG, Tuttlingen, Germany) connected to the microscope. A transplanum polare approach was performed and each salient step was described in detail with accompanying cadaveric pictures to improve understanding of the procedure. A video illustrating the cadaveric dissection in detail was also added (Video S1). A clinical case of cavernous hemangioma of the mesiotemporal lobe is also added including a surgical video of the procedure (Video S2).

## 3. Results

### 3.1. Topographic Anatomy of the Planum Polare and Its Surrounding Structures

**Figure 1:**
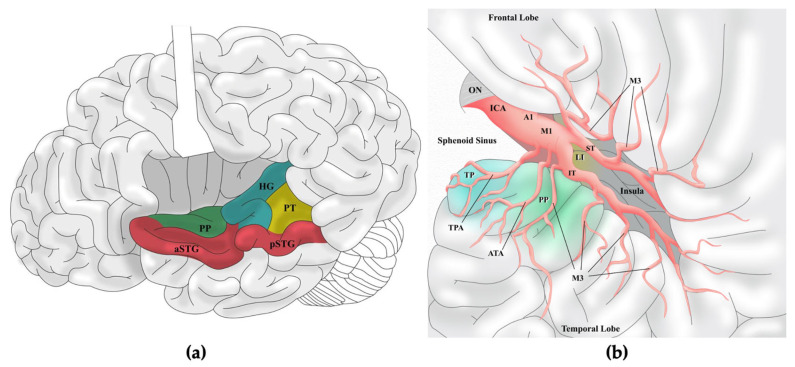
Anatomical landmarks and neurovascular relationships of the transplanum polare approach (TPPA). (**a**) Lateral view of the brain with the Sylvian fissure opened and the frontal operculum slightly retracted, highlighting the cortical anatomy of the superior temporal surface: the planum polare (PP) lies medially to the anterior portion of the superior temporal gyrus (aSTG) and anteriorly to the Heschl’s gyrus (HG), and the planum temporale (PT); (**b**) Graphic representation of a left-sided pterional approach with opened Sylvian fissure. The dissection reveals the PP in close relationship with the anterior temporal artery (ATA) and the anterior temporal M3 branches. PP is located significantly more superficially than the limen insulae (LI). A1: First segment of the Anterior Cerebral Artery; aSTG: anterior portion of the Superior Temporal Gyrus; ATA: Anterior Temporal Artery; HG: Heschl’s Gyrus; ICA: Internal Carotid Artery; IT: Inferior Trunk of the Middle Cerebral Artery; LI: Limen Insulae; M1: First segment of the Middle Cerebral Artery; M3: Third segment of the Middle Cerebral Artery; ON: Optic Nerve; PP: Planum Polare; pSTG: posterior portion of the Superior Temporal Gyrus; PT: Planum Temporale; ST: Superior Trunk of the Middle Cerebral Artery; TP: Temporal Pole; TPA: Temporopolar Artery.

The planum polare represents one of the most intricate and strategically significant regions of the temporal lobe, located on the superior surface of its anterior pole, immediately anterior to Heschl’s gyrus and in close relationship with the Sylvian fissure [13]. Medially, it is bounded by the uncus, which forms the most prominent landmark of the mesiotemporal region, embracing the hippocampal head and amygdala [14]. The uncus corresponds to the medial nucleus of the amygdala and serves as a key reference for neurosurgical approaches [15]. Beneath the cortical mantle of the planum polare and temporal pole lies the temporal stem, a compact bundle of white matter containing major associative pathways, as the uncinate fasciculus, linking the amygdala and hippocampus to the orbitofrontal cortex and playing a central role in emotional memory and semantic processing, and the inferior fronto-occipital fasciculus (IFOF), which connects occipital visual association areas with the frontal lobe and contributes to semantic cognition and visual integration [16,17]. The anterior commissure also traverses this region, mediating interhemispheric transfer of olfactory and limbic information. Importantly, the optic radiations, including Meyer’s loop, course through the temporal stem, sweeping anteriorly around the roof of the temporal horn before curving posteriorly toward the occipital lobe, illustrating the intimate relationship of visual pathways with anterior temporal structures [18]. Medially, the planum polare approaches the uncal and temporal pole regions, where the hippocampal head forms the medial floor and the amygdala occupies the anterosuperior wall of the temporal horn of the lateral ventricle [19]. The uncal recess separates these two structures, while the inferior choroidal point marks the entry of the anterior choroidal artery, a critical vascular landmark [20]. The surface of the planum polare contributes to forming the inferior wall of the Sylvian fissure, specifically the anterior portion of the temporal operculum, where the depression accommodates the anterior temporal artery (ATA) and the M3 segment of the middle cerebral artery (MCA) before its cortical branches (M4) [21]. This intimate vascular relationship underscores the surgical vulnerability of the area during anterior temporal approaches (Figure 1).

### 3.2. Step-by-Step Cadaveric Dissection

**Figure 2:**
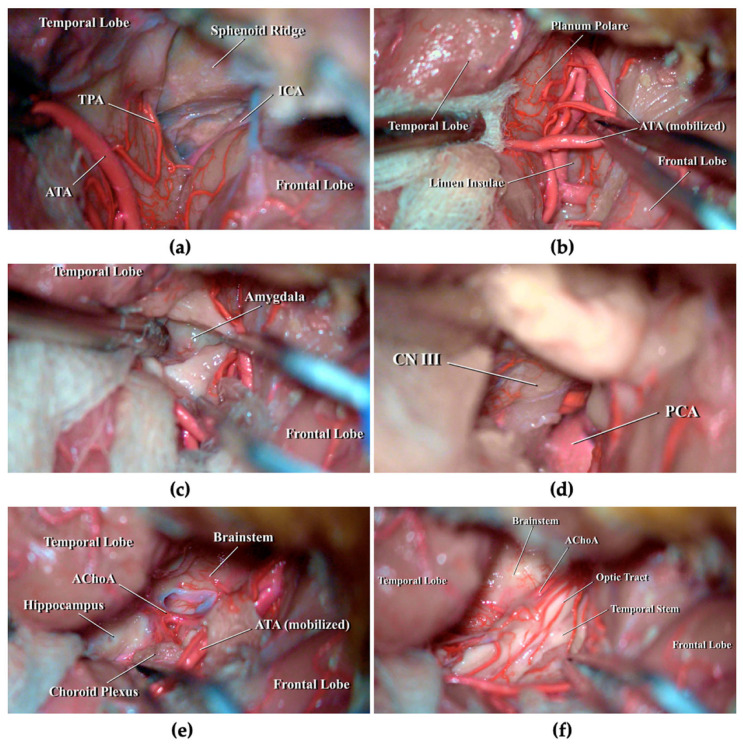
Stepwise cadaveric dissection of left TPPA. (**a**) After opening the Sylvian fissure, ATA and TPA branches of middle cerebral artery (MCA) were identified upon the superomedial surface of the temporal lobe; (**b**) Mobilization of ATA to expose the planum polare; (**c**) Identification of the amygdala after cortical incision of planum polare; (**d**) Resection of the amygdala and uncus exposing the PCA and the CN III; (**e**) Following PCA posteriorly within the temporal horn of the lateral ventricle, the dentate gyrus was removed exposing AChoA, choroid plexus and the anterior portion of hippocampus; (**f**) Superior and posterior portion of the amygdala were resected with exposure of the optic tract and the temporal stem. AChoa: anterior choroidal artery; ATA: anterior temporal artery; CN III: cranial nerve III; ICA: internal carotid artery; PCA: posterior cerebral artery.

Cadaveric dissection is demonstrated in Video S1. The head was positioned supine and secured in a three-pin Mayfield clamp, then rotated approximately 15° to the right and slightly extended. A skin incision was made along the superior border of the zygomatic arch near the tragus, extending posteriorly along the hairline toward the midline. After dissection of the temporal muscle, a left pterional craniotomy was performed, centered on the Sylvian fissure. A standard C-shaped dural opening provided adequate exposure of the frontal and temporal opercula, as well as the Sylvian fissure. The Sylvian fissure was carefully split to identify the temporopolar artery (TPA) and the ATA upon the superomedial surface of the temporal lobe (Figure 2a), until the planum polare was fully exposed. The ATA was cautiously mobilized to allow complete visualization of the planum, as a plain area located mesially to the anterior aspect of the superior temporal gyrus (STG) (Figure 2b). A 2 cm corticotomy was performed and the resection of the anteroinferior portion of the amygdala, together with the uncus, was carried out (Figure 2c) until the posterior cerebral artery (PCA) and cranial nerve III (CN III) were exposed (Figure 2d). The PCA was then followed posteriorly, removing the posterior portion of the uncus and the dentate gyrus, until the temporal horn of the lateral ventricle was reached. Along the medial ventricular wall, the choroid plexus and the inferior choroidal point were identified, marking the entry of the anterior choroidal artery (AChoA). Only the head of the hippocampus was accessible, as this approach generally limits exposure to the mid-mesencephalic level of the posterior mesiotemporal region (Figure 2e). Finally, removal of the posterosuperior part of the amygdala was completed, exposing the optic tract and the temporal stem (Figure 2f).

### 3.3. Clinical Case

**Figure 3:**
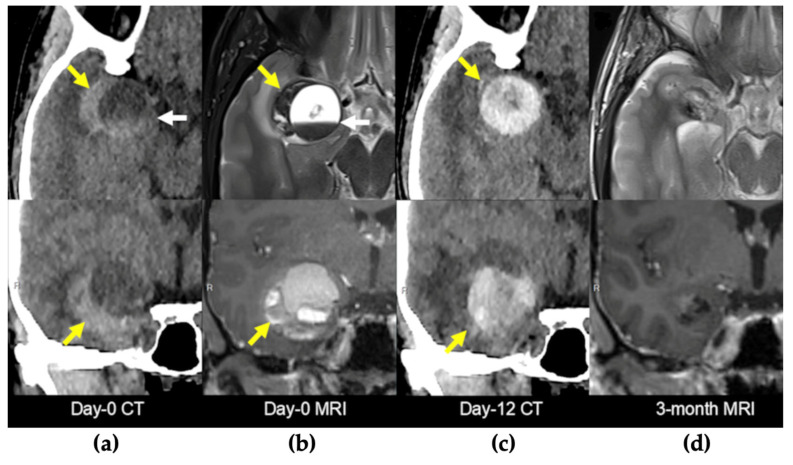
Neuroradiological findings. The upper row displays axial sequences, while the lower row shows coronal sequences. In (**b**,**d**), the upper row corresponds to a T2-weighted MRI, and the lower row to a contrast-enhanced T1-weighted MRI. (**a**,**b**) CT scan and MRI scan obtained at the patient’s initial admission showing a right temporopolar hematoma (yellow arrow) due to the rupture of a cavernoma containing a hemato-hematic level (white arrow). (**c**) CT scan performed upon readmission, 12 days after discharge, demonstrated new acute bleeding within the lesion (yellow arrow). (**d**) MRI performed three months after surgery showed small area of fibrotic tissue with no evidence of residual lesion or ischemic areas. CT: computed tomography; MRI: magnetic resonance imaging.

**Figure 4:**
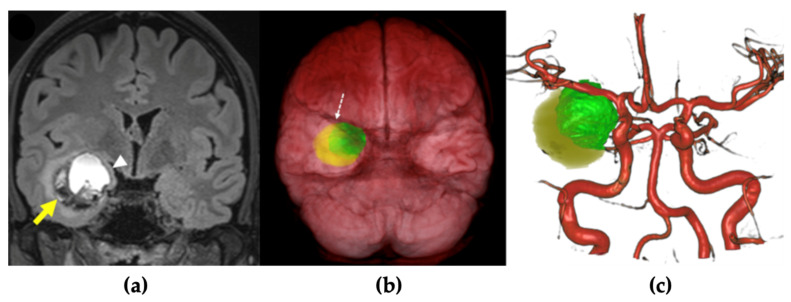
Preoperative MRI planning. (**a**) Preoperative coronal FLAIR-MRI demonstrated a large cavernomatous cavity filled with hyperintense blood products, along with a small hematoma located on its lateral and inferior aspects (yellow arrow). This caused compression and medial displacement of the right amygdala (white arrowhead). (**b**,**c**) 3D reconstruction and segmentation of the cavernoma (in green) and the hematoma (in yellow) showed that the lesion was situated immediately beneath the anterior temporal planum (white dashed arrow) in close proximity to the right middle cerebral artery.

**Figure 5:**
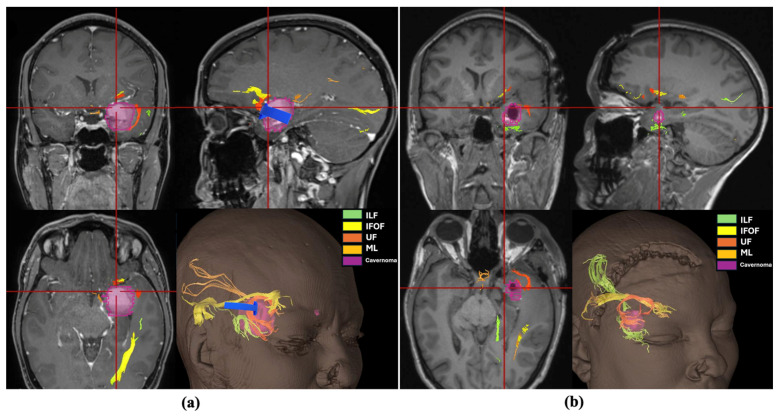
Three-dimensional reconstruction of the lesion (purple) and the surrounding major white matter tracts: inferior longitudinal fasciculus (ILF, green), inferior fronto-occipital fasciculus (IFOF, yellow), uncinate fasciculus (UF, orange), and Meyer’s loop (ML, golden yellow). The blue arrow indicates the direction of the surgical corridor and the entry zone. (**a**) Preoperative planning with definition of the anatomical relationships between the tumor and the white matter tracts, showing TPPA entry zone may avoid transgression of principal tracts. (**b**) Postoperative evaluation showing preservation of the major white matter tracts after complete resection. IFOF: inferior fronto-occipital fasciculus; ILF: inferior longitudinal fasciculus; ML: Meyer’s loop; UF: uncinate fasciculus.

A 27-year-old female patient with a history of occasional migraine episodes (1–2 per year, treated with triptans) was admitted following a car accident. She experienced a brief loss of consciousness preceded by facial paresthesia and migraine-like symptoms, without urinary incontinence or tongue biting. Upon regaining consciousness, she exhibited circumstantial amnesia but no external signs of cranial trauma. Neuroimaging (CT and MRI) revealed a right intraparenchymal hematoma with mixed acute and subacute components located in the mesiotemporal region (Figure 3a,b). The patient was managed conservatively and discharged on antiepileptic therapy with levetiracetam. A follow-up brain MRI was scheduled for two months later. Twelve days after discharge, the patient presented again with severe headache. She reported a recent increase in the frequency of migraine episodes, now accompanied by prodromal nasal tingling and near-syncope, followed by fatigue after each episode. A new CT scan revealed fresh acute bleeding within the lesion (Figure 3c). Given the patient’s recurrent, disabling neurological episodes and worsening headaches unresponsive to medical therapy, surgical treatment was indicated. Preoperative neuroradiological evaluation revealed a large cavernomatous cavity filled with hyperintense blood products, along with a small hematoma on its lateral and inferior aspects. The lesion was located immediately beneath the anterior temporal planum and in close proximity to the middle cerebral artery (MCA) (Figure 4). The lesion was completely removed via a transplanum polare approach (Video S2). No intraoperative complications were encountered. Postoperative MRI confirmed complete resection of the lesion. Tractography showed preservation of the major white matter tracts surrounding the lesion after its removal (Figure 5). The patient exhibited no neurological deficits after surgery and was discharged four days postoperatively. At the three-month follow-up, she remained neurologically intact, with a well-healed surgical wound and no evidence of residual or ischemic lesions on MRI (Figure 3d).

## 4. Discussion

The Transplanum Polare Approach (TPPA) is a microsurgical route to the mesiotemporal region that uses the planum polare as an entry point, providing a direct trajectory to the amygdala and hippocampal head [13]. While transsylvian approaches to the mesiotemporal region have already been reported, direct access through the planum polare has been only previously described by Starnoni et al. [13]. Accordingly, the novelty of the present study does not reside in proposing an entirely unprecedented corridor, but rather in more precisely characterizing the planum polare as a dedicated entry zone and in discussing its potential anatomical and technical advantages with respect to the existing approaches to the mesiotemporal region [22]. More specifically, we propose this route as a potentially safer alternative to the classical transsylvian translimen approach, as it may reduce the need for extensive Sylvian fissure dissection and thereby lessen the risk of vascular injury or vasospasm. At the same time this corridor may also potentially help limiting the transgression of the temporal stem and Meyer’s loop with respect to the classic transsylvian translimen approach, although our observations are based on a single clinical case and cannot be generalized. We also acknowledge that, in practice, some neurosurgeons performing transsylvian approaches may already reach the mesial temporal region through an entry point close to the planum polare, particularly depending on the single vascular anatomy that may render more complex or hazardous the exposure of the limen insulae. However, this entry zone has not been clearly identified, anatomically characterized, and specifically discussed so far.

### 4.1. TPPA vs. Transcortical Approach

Transcortical approaches remain among the most widely used techniques, particularly in epilepsy and tumor surgery, as they provide broad exposure of the hippocampus and amygdala [23]. However, they require a cortical incision through the middle or inferior temporal gyrus, possibly affecting associative and visual pathways within the temporal stem and the roof of the temporal horn [24]. The Meyer’s loop, which represents the anterior sweep of the optic radiations, is particularly vulnerable, and its injury typically results in contralateral superior quadrantanopia [25]. Likewise, disruption of the IFOF, uncinate fasciculus, or anterior commissure may lead to deficits in semantic processing, memory encoding, emotional regulation, and language fluency [26]. In contrast, the TPPA provides an anterobasal trajectory to the anterior temporal horn, avoiding cortical incision and possibly minimizing the disruption of temporal stem fibers. Entering the ventricle medial to Meyer’s loop could indeed reduce the risk of visual field deficits and preserves major associative pathways, thereby offering a clear functional advantage for anterior mesiotemporal lesions (Table 1).

### 4.2. TPPA vs. Transsylvian Translimen Approach

The transsylvian translimen approach avoids cortical incision and offers a cisternal route to the mesiotemporal region. However, it requires a wide opening of the Sylvian fissure and mobilization of the M2 MCA segments [27]. This extensive dissection increases the risk of injury to deep perforators supplying the internal capsule, basal ganglia, and optic tract, which can result in severe motor or visual deficits [18]. Furthermore, the translimen approach may lead to temporal stem transgression, leading to language impairment, memory dysfunction, and superior quadrantanopia [28]. TPPA requires minimal manipulation of MCA branches, thereby preserving the integrity of both deep perforators and major associative tracts. The planum polare lies posterolaterally to the limen insulae, thus rarely requiring mobilization of the more distal MCA branches. This anatomic relationship makes the TPPA a posterior-to-anterior and superior-to-inferior surgical corridor, limiting exposure primarily to the anterior mesiotemporal region. This particular corridor may reduce the risk of injury of the temporal stem and Meyer’s loop with respect to the classic transsylvian translimen approach even though further clinical data are requested to corroborate this statement (Table 1).

### 4.3. TPPA vs. Subtemporal Approach

The subtemporal approach, on the other hand, provides excellent visualization of the posterior mesiotemporal lobe. Temporal lobe retraction allows direct exposure of the fusiform gyrus and entry into the temporal horn through its anteroinferior wall, creating an anteroposterior surgical trajectory [29]. This enables good visualization of the posterior hippocampus while sparing the temporal neocortex, temporal stem, and Meyer’s loop [6]. However, the required retraction may injure the basal temporal veins and potentially affect basal temporal language areas, especially on the dominant hemisphere [30]. Moreover, the landmarks for entering the temporal horn via the subtemporal route are not always distinct and often rely heavily on neuronavigation, which can be distorted by brain shift after dural opening, increasing the risk of iatrogenic injury [6]. While the TPPA does not provide adequate exposure of the posterior hippocampus, it may be a safe and effective alternative for treating pathologies localized to the anterior hippocampus. This is especially true for the dominant hemisphere, as the TPPA eliminates the need for static temporal lobe retraction the risk of injuring the Vein of Labbé (Table 1).

### 4.4. TPPA vs. Paramedian Supracerebellar–Transtentorial Approach

The paramedian supracerebellar–transtentorial (PST) approach, first described by Türe et al. in 2012, provides a direct and safe route to the mesial temporal structures through a medial corridor to the collateral sulcus, thereby avoiding neocortical transgression [31]. This approach enables highly selective resection of meso- and allocortical components, minimizing manipulation of healthy tissue and offering excellent intraoperative orientation with clearly defined anatomical boundaries. Compared with transcortical and transsylvian routes, the PST approach allows complete removal of the hippocampal formation—from the uncus to the level of the inferior colliculus—without extensive corticectomy or brain retraction. The main limitations are related to the semisitting position, namely potential surgeon discomfort and the risk of air embolism. However, in selected cases, the approach can be safely performed in the lateral position without compromising surgical exposure. Moreover, the PST approach demands advanced anatomical knowledge and refined microsurgical skills, as the surgical corridor is narrow and deep, extending from the posterior cranial fossa to a target located in the middle cranial fossa [9]. As with the comparison to the subtemporal approach, the TPPA is less effective than the PST approach for removing the posterior portion of the hippocampus. Nevertheless, for lesions that do not involve the posterior mesiotemporal region, we believe the TPPA represents a highly viable option: it appears to be technically more straightforward, less time-consuming, and physically less demanding for the surgeon compared to the PST approach (Table 1).

### 4.5. TPPA vs. Endoscopic Approaches

In recent years, the advent of endoscopic surgery has enabled surgeons to approach the anterior temporal pole through less invasive corridors. The mesial temporal lobe can also be accessed via transoccipito-ventricular, transorbital, or endonasal routes.

The transorbital approach provides a direct and wide exposure of the mesial temporal lobe but carries potential risks of orbital injury, periorbital scarring, or diplopia due to globe retraction. Cerebrospinal fluid (CSF) leakage may also occur depending on the extent of skull base resection and orbital reconstruction required [10,11]. In comparison, the endonasal endoscopic approach (EEA) generally allows safer manipulation with less risk to adjacent structures. The EEA through the V2–V3 corridor reaches the mesial temporal lobe along a more medial and superior trajectory, requiring unnecessary exposure of the pterygopalatine fossa and partial disruption of the lateral neocortex [32]. In contrast, the V1–V2 corridor offers a shorter, more direct route to the target, minimizing parenchymal and orbital injury. Nevertheless, this route carries risks of V1–V2 injury, which may cause facial numbness, neuropathic pain, diplopia due to abducens palsy, or dry eye from zygomatic nerve sacrifice, potentially leading to corneal complications [33]. While endoscopic techniques (transorbital and endonasal routes) are inherently minimally invasive, their specific surgical corridors carry unique risks that must be weighed against patient-specific factors, such as anatomical variants or concomitant pathologies in these regions. Furthermore, endoscopic approaches generally offer more limited exposure compared to transcortical routes; therefore, the extent of the lesion is a critical factor in surgical planning. In this landscape, the TPPA could represent a less invasive alternative to other transcranial surgical approaches while effectively eliminating the specific risks associated with endoscopic corridors and maximizing surgical exposure for anterior mesiotemporal region (Table 1).

### 4.6. TPPA Disadvantages

Despite its potential advantages, TPPA has some intrinsic drawbacks. First, it offers a relatively restricted operative corridor, which may not be adequate for large lesions, lesions with marked posterior extension, or lesions involving the temporal stem. Exposure of the posterior mesiotemporal region rarely extends beyond the mid-mesencephalic level, thereby limiting visualization and safe resection of lesions involving the hippocampal body, tail, or atrium. In such situations, the narrowness of the approach may restrict surgical freedom and increase the risk of blind dissection or excessive retraction as well as potential risk of temporal stem transgression. Therefore, lesions extending to the posterior mesiotemporal region or involving the temporal stem may be more appropriately managed through alternative corridors, such as the subtemporal or posterior transcortical approaches, which offer a broader and more direct operative window [34] (Table 1).

In addition, anatomical variability of the Sylvian fissure and specific vascular anatomy may further complicate exposure in selected cases.

## 5. Limitations

This study is subject to several limitations. First, the use of a single cadaveric dissection precludes the illustration of anatomical variations that might complicate this approach in a broader population. This dissection was intended solely to provide a descriptive representation of the proposed technique. Furthermore, structured anatomical comparisons and quantitative measurements are required to objectively evaluate the advantages of this approach over existing surgical alternatives. Additionally, a single clinical case is insufficient to draw definitive conclusions regarding superior clinical benefits, and it is intended to be merely illustrative of the surgical technique.

## 6. Conclusions

The transplanum polare approach represents a novel, safe, and functionally advantageous route to the mesiotemporal region. By exploiting an anterobasal trajectory through the planum polare, this approach minimizes cortical transgression, Sylvian fissure dissection, and manipulation of major MCA branches, thereby reducing the risk of injury to eloquent cortical areas, associative fibers, and deep perforators. Compared with traditional transsylvian, transcortical, or subtemporal routes, TPPA may offer a more direct trajectory to the anterior temporal horn while potentially reducing manipulation of the temporal stem and adjacent white matter pathways, including Meyer’s loop. However, the impact of this anatomical corridor on functional outcomes remains to be established. Despite its limited working corridor and reduced exposure of posterior mesiotemporal structures, the integration of modern microsurgical techniques, preoperative tractography, and intraoperative navigation can enhance precision and safety. Overall, the TPPA provides a valuable addition to the microsurgical armamentarium for mesiotemporal lesions, combining minimal invasiveness with maximal preservation of neurovascular and functional integrity.

## Figures and Tables

**Table 1:** Comparative overview of surgical approaches to the mesiotemporal lobe. CSF: cerebrospinal fluid; MCA: middle cerebral artery.

Approach	Entry Point	Advantages	Disadvantages
Transplanum Polare	Planum Polare	Direct anterobasal route Avoids wide Sylvian fissure dissection and lateral cortex incision Uses standard pterional craniotomy	Limited access to posterior hippocampus Anatomical variability of planum polare and Sylvian fissure
Transcortical	Middle/Inferior Temporal Gyrus	Wide exposure Direct and relatively simple trajectory No need for vascular manipulation	Requires cortical incision Risk of language and memory deficits (dominant hemisphere) Risk of visual field defects (Meyer’s loop)
Transsylvian/Translimen	Limen Insulae	Avoids cortical incision Preserves lateral neocortex Direct access to amygdala and anterior hippocampus	Technically demanding Narrow surgical corridor Risk to MCA branches and perforators Limited exposure to posterior hippocampus.
Subtemporal	Parahippocampal Gyrus	Wide exposure Avoids the lateral temporal cortex, the Sylvian fissure and the temporal stem No need for vascular manipulation	Requires significant temporal lobe retraction Risk of venous infarction (vein of Labbé)
Supracerebellar Transtentorial	Parahippocampal Gyrus	Direct route to the mesial temporal structures Clearly defined anatomical boundaries No neocortical transgression or brain retraction	Semisitting position (surgeon discomfort, risk of air embolism) Narrow and deep surgical corridor
Endoscopic Endonasal	Parahippocampal Gyrus	Direct access to mesial structures without traversing lateral neocortex No brain manipulation Excellent cosmetic outcome	Limited access to posterior hippocampus Risk of CSF leak, infection, and sinonasal morbidity Technically complex
Endoscopic Transorbital	Temporal Pole	Direct access to mesial structures without traversing lateral neocortex No brain manipulation Excellent cosmetic outcome	Narrow and depth surgical corridor Limited access to posterior hippocampus Risk to orbital structures Risk of CSF leak

## Data Availability

The original contributions presented in this study are included in the article. Further inquiries can be directed to the corresponding author.

## Supplementary Materials

The following supporting information can be downloaded at: https://www.mdpi.com/article/10.3390/brainsci16040351/s1, Video S1: Cadaveric Dissection; Video S2: Intraoperative Video.

## Abbreviations

The following abbreviations are used in this manuscript:

2D/4K	Two-Dimensional/4K-Resolution
A1	First segment of the Anterior Cerebral Artery
AChoA	Anterior Choroidal Artery
aSTG	anterior Superior Temporal Gyrus
ATA	Anterior Temporal Artery
CN III	Cranial Nerve III
CSF	Cerebrospinal Fluid
EEA	Endoscopic Endonasal Approach
HG	Heschl’s Gyrus
ICA	Internal Carotid Artery
IFOF	Inferior Fronto-Occipital Fasciculus
ILF	Inferior Longitudinal Fasciculus
IT	Inferior Trunk of the Middle Cerebral Artery
LI	Limen Insulae
M1	Horizontal Segment of the Middle Cerebral Artery
M2	Insular Segment of the Middle Cerebral Artery
M3	Opercular Segment of the Middle Cerebral Artery
M4	Cortical Branches of the Middle Cerebral Artery
MCA	Middle Cerebral Artery
ML	Meyer’s Loop
NETLab	Neurosurgical Education and Training Laboratory
ON	Optic Nerve
PCA	Posterior Cerebral Artery
PP	Planum Polare
pSTG	posterior Superior Temporal Gyrus
PT	Planum Temporale
ST	Superior Trunk of the Middle Cerebral Artery
TP	Temporal Pole
TPA	Temporopolar Artery
TPPA	Transplanum Polare Approach
UF	Uncinate Fasciculus
V1	Ophthalmic Branch of the Trigeminal Nerve
V2	Maxillary Branch of the Trigeminal Nerve
V3	Mandibular Branch of the Trigeminal
